# Potential of Plantain Pseudostems (*Musa AAB Simmonds*) for Developing Biobased Composite Materials

**DOI:** 10.3390/polym16101357

**Published:** 2024-05-10

**Authors:** Juan Pablo Castañeda-Niño, Jose Herminsul Mina Hernandez, Jose Fernando Solanilla Duque

**Affiliations:** 1 Grupo Materiales Compuestos, Escuela de Ingeniería de Materiales, Universidad del Valle, Calle 13 No. 100-00, Cali 76001, Colombia; juan.castaneda.nino@correounivalle.edu.co; 2 Departamento de Agroindustria, Facultad de Ciencias Agrarias, Universidad del Cauca, Sede Las Guacas, Popayán 190001, Colombia; jsolanilla@unicauca.edu.co

**Keywords:** sap, lignocellulosic fibers, plantain starch, steam explosion, biorefineries, plantain pseudostems

## Abstract

A plantain pseudostem was harvested and processed on the same day. The process began with manually separating the sheaths (80.85%) and the core (19.14%). The sheaths were subjected to a mechanical shredding process using paddles, extracting 2.20% of lignocellulosic fibers and 2.12% of sap, compared to the fresh weight of the sheaths. The fibers were washed, dried, combed, and spun in their native state and subjected to a steam explosion treatment, while the sap was subjected to filtration and evaporation. In the case of the core, it was subjected to manual cutting, drying, grinding, and sieving to separate 12.81% of the starch and 6.39% of the short lignocellulosic fibers, compared to the fresh weight of the core. The surface modification method using steam explosion succeeded in removing a low proportion of hemicellulose and lignin in the fibers coming from the shims, according to what was shown by Fourier Transform Infrared Spectroscopy (FT-IR), Thermogravimetric Analysis (TGA), and Differential Scanning Calorimetry (DSC), achieving increased σ_max_ and ε from the tensile test and greater thermal stability compared to its native state. The sap presented hygroscopic behavior by FT-IR and the highest thermal stability from TGA, while the starch from the core presented the lowest hygroscopic character and thermal stability. Although the pseudostem supplied two types of fibers, lower lignin content was identified in those from the core. Finally, the yarns were elaborated by using the fibers of the sheaths in their native and steam-exploded states, identifying differences in the processing and their respective physical and mechanical properties.

## 1. Introduction

The cultivation of the plantain variety Dominico Hartón has contributed to the economy of the actors belonging to its agro-chain (farmers, processors, and distributors) in tropical countries, such as Colombia, through the sale and processing of the bunch for the use of the pulp [1,2,3], which contributes to human food through its fresh consumption in the form of flour or snacks. Additionally, in the animal feed sector, concentrates have been developed [3,4] that contain a plantain biomass of between 12 and 20% [3,4,5], while the other by-products found in the mother plant have been considered as “waste” [6,7] or have been used at the commercial level as raw material in some simple processing related to the development of organic fertilizers (by composting the leaves, rachis, acorn, and pseudostem) and the creation of handmade fabrics from the long fibers of the pseudostem [1,2,3,5,8]. The above practices have not taken advantage of the potential of the plantain crop, through the identification of molecules and macromolecules available in by-products, such as pseudostems, to be able to be processed into transformed products, thus generating added value in a respective production chain. The plantain pseudostem consists of a set of overlapping sheaths organized in a spiral around the core or medulla, containing a moisture content of 93.2 to 96.0%. Each sheath contains lignocellulosic fibers in a longitudinal orientation [8,9,10] and a composition based on cellulose (56 to 60%), hemicellulose (43%), lignin (19 to 20%), pectin, waxes, and water-soluble substances [9,11,12]. The core has a cylindrical geometry with an approximate diameter between 5 and 6 cm and is another edible component of the plantain; it could be possible to identify culinary alternatives in India and Malaysia that use this by-product for human consumption, since it contains fibers and starch with a yield between 0.12 and 1.00% [1,12,13,14]. Another component of interest is found in the pseudostem’s sheaths and core, namely the plantain sap, which is a highly available powdery brown liquid that represents 90% of the moisture contained in the pseudostem, since 1500 to 20000 L of sap can be obtained in one hectare of plantain, and it is composed of proteins, carbohydrates, and fibers [15], as well as tannin-based compounds, phenols, and aromatic compounds [16,17]. Sap can be a stable liquid at a wide pH range, achieving a colorless appearance at acidic pH, while a brown color is maintained at alkaline pH [18,19].

Currently, the above by-products identified in plantain pseudostems have fulfilled some applications of interest, such as paper making and power generation [1,12]; reinforcement in concretes [10,20] and polymers [3,11,12,21]; obtaining nanocrystals for reinforcement in composites and biosorbents [22,23]; making textiles and panels for interiors [11,12]; bioethanol and biogas production [3]; infusion bag production [8]; production of diapers, yarns, threads, twines, and ropes [24,25]; creation of dyes and flame-retardant additive for textiles [16]; and production of functional beverages with antioxidant properties that can treat symptoms of diseases, such as flu, cough, diabetes, cancer, and HIV, among others [25,26].

In the present research, pseudostems from the plantain variety Dominico Hartón were used to obtain lignocellulosic fibers, starch, and sap using a mechanical pallet shredder for physicochemical, thermal, structural, and morphological characterization, as well as to perform surface modification of lignocellulosic fibers from pseudostem sheaths. This was followed by the development of yarn from the native and modified long fibers, characterizing them at a physical and mechanical level (tension test) in order to evaluate the above products made from the plantain pseudostem as possible reinforcements (fibers and yarns) and coatings (sap) to reduce water absorption in a biobased composite based on thermoplastic plantain starch.

## 2. Materials

Plantain pseudostems of the Dominico Hartón variety were harvested between the 17th and 18th week after the beginning of flowering in Villa Rica, Cauca (altitude: 948 m.a.s.l.) through the Asociación de Productores de Finca Tradicional del Norte del Cauca (ASPROFINCA).

## 3. Experimental Procedure

### 3.1. Extraction of Lignocellulosic Fibers, Sap, and Starch from Plantain Pseudostems

The fibers were extracted on the same day the pseudostem was harvested to avoid decomposition processes in the respective tissues. Initially, each of the sheaths and the core was removed manually, followed by the defibration of each sheath using a mechanical shredder with a paddle rotor with a speed of 466 r.p.m. and a thickness of 4.15 mm at the entrance of the shredder. Long lignocellulosic fibers and a cake were obtained at the end of the shredding process, requiring pretreatment in order to condition the two resulting by-products. In the case of native lignocellulosic fibers (FNSP), they were subjected to washing with distilled water to remove some sap and parenchyma residues that may have been retained, followed by forced convection drying at a temperature of 60 °C for 12 h, and they were finally stored in high-density polyethylene (HDPE) bags and stored in a dark place [27,28]. In the case of the cake, manual compression was performed to separate, obtain, and store the sap (SSP) in HDPE bags, followed by storage under frozen conditions (−20 °C). When the plantain sap was obtained, it was dried using a forced convection oven at 70 °C for 24 h. The plantain pseudostem core was chopped to obtain a “chip” presentation to initiate its drying by forced convection (60 °C for 24 h), followed by milling using a knife mill considering a 2 mm sieve. The flour from milling was subjected to sieving, separating the short lignocellulosic fibers (FNCO) and starch (ASP) using the 100th sieve according to the Tyler series.

#### 3.1.1. Steam Explosion

The surface modification of the plantain pseudostem fibers (FMSP) was carried out using the steam explosion methodology [29], where the fibers were introduced into an autoclave subjecting them to a temperature of 120 °C and a pressure of 0.15 MPa for 30 min, followed by instant decompression. Subsequently, the fibers were immersed in distilled water for 30 min, followed by forced convection drying at 70 °C for 12 h. Alkalized sheath fibers, resulting from immersion in a 2% sodium hydroxide solution at 80 °C for 1 h, were used as a standard for comparison.

#### 3.1.2. Yarn Production from Lignocellulosic Fibers

Once the native and modified lignocellulosic fibers from the plantain pseudostem were available, they were combed to remove possible knots. Subsequently, a mechanical braiding process was carried out to achieve yarns with a diameter of approximately 2 mm [27].

### 3.2. Cellulose, Hemicellulose, and Lignin Content in Plantain Fibers

With some modifications, Klason lignin was determined according to Tappi 222- om88 [30]. For this purpose, extractable and water-soluble free fibers (300 mg) were hydrolyzed with 72% (*w*/*w*) sulfuric acid at 30 °C for 1 h in pyrex flasks. Subsequently, the solutions were diluted with distilled water to a 4% sulfuric acid concentration and kept for 1 h at 110 °C. Once cooled, they were filtered through porous glass filters (Vidra FOC 663/3), previously tared (4 h at 100 °C). The first 100 mL of the filtrates were collected for subsequent analysis of free sugars. Klason lignin was retained on porous glass filters. The residue (Klason lignin) was washed with distilled water to a neutral pH and dried at 100 °C for 4 h. The percentages of Klason lignin were calculated by weight difference. The ash content of Klason lignin was determined using Tappi 211 om-85 [31]. Such content was estimated from the total percentage of lignin in the samples [32]. Holocellulose, hemicellulose, and cellulose-α were determined from the TAPPI T203, 1999 standard [20,33], considering Equation (1).

(1) Hemicellulose=(holocellulose−α cellulose)

### 3.3. Physical, Thermal, and Mechanical Properties of Fibers, Starch, and Sap from Plantain Pseudostems

#### 3.3.1. Fourier Transform Infrared Spectroscopy (FT-IR)

An FT-IR spectrophotometer Shimadzu, IRAffinity-1 (Kansai, Kyoto, Japan) with an attenuated total reflectance (ATR) accessory was used. These experiments were performed according to ASTM E1252. The samples were kept at 50% relative humidity and 23 °C, while the analysis was performed at 100 scans and in a range between 4000 and 550 cm^−1^ [34].

#### 3.3.2. Scanning Electron Microscopy (SEM)

Morphological analysis of the samples was performed on a scanning electron microscope JEOL, JSM 6490 LV (Jeol, Mexico D.F., Mexico). The samples were coated with a palladium gold layer. One milligram of each plantain pseudostem by-product was used to be placed on a carbon ribbon. The images obtained were from the backscattered electron method with an accelerating power of 20 kV and a vacuum of 30 Pa in the microscope chamber, achieving magnifications of 500 and 1000× [14].

#### 3.3.3. Thermogravimetric Analysis (TGA)

Thermogravimetric analysis equipment TA Instruments Q50 (New Castle, DE, USA) was used to study the thermal stability of the pseudostem by-product. The weight of the samples was kept between 5.0 and 8.0 mg. Each sample was subjected to a temperature range from 25 to 600 °C at a heating rate of 10 °C/min in a nitrogen atmosphere (analytical grade 5.0) with a heat flow of 50 mL/min. The values obtained were the percentage of moisture, weight loss, and decomposition temperature (Td) from the derivative of the TGA curve (DTGA) [32].

#### 3.3.4. Differential Scanning Calorimetry (DSC)

10 mg was used in each sample, which had been previously conditioned at a relative humidity of 50% and a temperature of 23 °C and evaluated using a calorimeter TA Instruments DSC25 (New Castle, DE, USA) [29,35]. Each of the samples was placed inside an airtight aluminum capsule, sealed, and placed inside the thermal chamber of the DSC. In an inert environment with nitrogen, the first heating cycle, from room temperature to 90 °C, was performed to erase the thermal history, followed by an isotherm of 90 °C for 5 min. This was followed by a cooling cycle from 90 °C to −60 °C and a −60 °C isotherm for 5 min. Finally, a heating cycle from −60 to 250 °C was performed to determine the glass transition temperature (Tg), melting temperature (Tm), and enthalpy (ΔHm) in the respective samples. The heating rate was 10 °C/min.

#### 3.3.5. Diameter and Linear Density of Fibers and Yarns

Diameter and density were estimated for the extracted fibers and the plantain pseudostem yarn by using a stereoscope coupled to a digital camera Nikon SMZ1000 (Styria, Graz, Austria) and the gravimetric method of ASTM D1577, 2018 [36] (atmospheric conditions), respectively. In determining diameter, 25 measurements were taken during each treatment using a magnitude of 10×. During the determination of the linear density (D), 25 samples were evaluated, placed on a flat surface to perform their cut, and considered a template to establish a standard length (L), after having been cut and weighed (W). The linear density of the samples was calculated through the model presented in Equation (2).

(2) $$D=\frac{10000\times W}{\left(L\times N\right)}$$

where *N* corresponds to the number of fibers in the sample, *L* is the length of the fibers in mm, and *W* is the weight of the sample in mg.

#### 3.3.6. Tensile Mechanical Tests

The tensile mechanical properties of the fibers and yarn (native and modified) were determined using ASTM D3822 [37]. Initially, the specimens were conditioned at a relative humidity (RH) of 50% and a temperature of 23 °C for 48 h. The specimens’ dimensions (apparent diameter) were determined using a stereoscope (Nikon SMZ1000) and image analysis software (S-Eye (S-EYE_Setup-1.6.0.11)). A universal testing machine Tinius Olsen H50Ks (Horsham, PA, USA) was used, taking into consideration the following conditions: 12.7 mm length between jaws, moving jaw displacement speed of 4.0 mm/min, a sample length 1.5 times longer than the distance between grips, and use of fiber support to guarantee its alignment between grips and protect it from any mechanical action that could cause it to fracture or twist. With the load and displacement information of the equipment, the maximum tensile strength (σ_max_), strain at the breaking point (ε), modulus of elasticity (E), and toughness (N/tex) were obtained [5,12].

## 4. Results and Discussions

### 4.1. Extraction of Lignocellulosic Fibers, Sap, and Starch from Plantain Pseudostems

**Figure 1:**
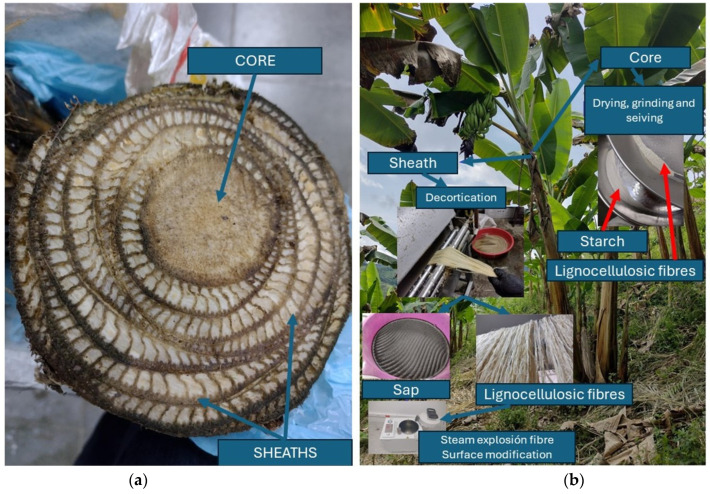
Types of pseudostem tissue and by-products. (**a**) sheaths and core and (**b**) plantain tissues.

The pseudostem, a plantain by-product, has two types of tissues, the sheaths and the core (see Figure 1a), comprising 80.85 and 19.14%, respectively (see Table 1), and containing a moisture content of 91.25 + 0.13%. According to Cadavid et al., 2016 [11], the content of cortex in plantain pseudostems can reach values of 26%, while the water content present in these tissues was found within the identified range compared to that reported in banana and plantain pseudostems with values between 83.0 and 94.6% [2,9,11,38].

In the chemical composition of both plantain tissues, lignocellulosic fibers are found (see Figure 1b), with the presence of 2.20% of long fibers in the sheaths, while 6.39% of short fibers (<4 mm) were identified in the core. The fiber content in the plantain sheaths was higher than that obtained by Cadavid et al., 2016, who reported an average yield of 1.10% of fibers from the sheaths and did not consider the amount of fibers present in the core since, in their attempt to extract them by using the mechanical shredder, their breakage was evidenced. In each tissue, a second characteristic component was identified, finding plantain sap mainly in the sheaths with 2.12% and starch in the core with 12.81% (see Table 1). This value is similar to that obtained by Shantha and Siddappa, 1970 [39], who indicated a yield of 10.2%. It is worth mentioning that, when the lignocellulosic fibers were extracted and separated from the plantain sap, reduced portions of starch, which did not exceed 1% of the weight of the sheaths, were identified. Jain et al., 1956 [13], reported starch contents in banana pseudostems (sheaths and core) of between 1 and 4% (b.h.). In the case of Subrahmanyan et al., 1957 [40], they found a range of between 4.5 and 5.0% at the time of harvesting the plantain bunch, while Mohd Ali et al., 2021 [14], identified a content between 0.12 and 0.20% in the banana variety Pisang Boyan, considering that values obtained below 1% do not generate any economic interest in its extraction [13]. According to the above, and identifying the starch yield in the core while considering the weight of the entire pseudostem, a value of 2.45% (b.h.) was obtained, which is within the range suitable to generate economic interest, recommending what was mentioned by Shantha and Siddappa, 1970 [39], and Subrahmanyan et al., 1957 [40], where the pseudostem should be harvested and processed in the shortest possible time to avoid losses in starch yields, since it hydrolyzes as the post-harvest storage time progresses. Considering the previous extraction yields of the byproducts, in the plantain’s mother plant pseudostem, an average of 203.66 g of sheath fiber, 290.67 g of sap, 238.61 g of core starch, and 81.15 g of core fiber can be obtained.

### 4.2. Cellulose, Hemicellulose, and Lignin Content in Plantain Fibers

The chemical composition of lignocellulosic fibers from musaceae (plantain, banana, and abaca) pseudostem sheaths is based on cellulose contents ranging from 31.27 to 69.09%, hemicellulose ranging from 10.00 to 28.00%, and lignin ranging from 5.00 to 19.13% [41,42,43,44,45,46,47]. The evaluated native fibers of the Dominico Hartón variety had a value of 54.72% cellulose, 13.92% hemicellulose, and 27.68% lignin, finding cellulose and hemicellulose contents within the ranges reported in other investigations (see Table 2). In contrast, the lignin content was higher, with values close to those given by timber species (22 to 30%) but lower than that generated in coconut fibers, with values between 40 and 45% [46]. Likewise, when comparing the chemical composition of the native plantain fiber sample (see Table 2) with other reports found in plantains, a low cellulose content (56.83 to 69.49%), adequate hemicellulose content (11.40 to 11.85%), and high lignin content (13.17 to 19.13%) were presented [41,42,47].

On the other hand, using different surface modification methods on the fibers contributed to reducing lignin content. In this sense, the steam explosion method generated a slight decrease in this macromolecule due to the use of water vapor at high temperature and pressure, which is a procedure of lower intensity compared to that achieved by alkalinization (see Table 2), allowing the removal of lignin and hemicellulose in more significant proportions due to the high pH and high temperature employed (see Table 2) [41,48]. Periyasamy et al., 2022, and Agbor et al., 2011, mentioned that steam explosion removes mainly hemicellulose and a reduced portion of lignin. However, only a slight reduction in lignin content was evidenced in the steam-exploded plantain pseudostem fibers. In contrast, hemicellulose content remained unchanged under steam explosion conditions despite increased standard deviation. Cardona Alzate et al., 2019 [48], proposed that an optimal surface modification of lignocellulosic fibers could be generated by employing temperatures between 160 and 290 °C and pressures between 0.7 and 4.85 MPa during the execution of steam explosion. In the case of lignin content, its value presented a slight reduction, which was a characteristic of interest for the elaboration of bio-based composite materials with higher values in mechanical properties since it can generate a mechanical anchorage and the formation of hydrogen bridges in the interfacial zone formed between the fibers with the presence of cellulose (reinforcement) and the polymeric matrix (depending on its nature) [49]. In the case of native fibers coming from the core, the lignin content was determined to be lower compared to that evidenced in the fibers coming from the sheaths (see Table 2). This was because the presence of lignin in the fibers was reduced as they are located in the sheaths that constitute the middle and internal structure of the pseudostem, this happens because the plant cells that constitute the sheaths in the external layer have a higher degree of maturity [12]. Mohapatra et al., 2009 [50], reported a lignin content of 6% in the pseudostem core of plantain, a value close to that obtained in this research.

### 4.3. Fourier Transform Infrared Spectroscopy (FT-IR)

**Figure 2:**
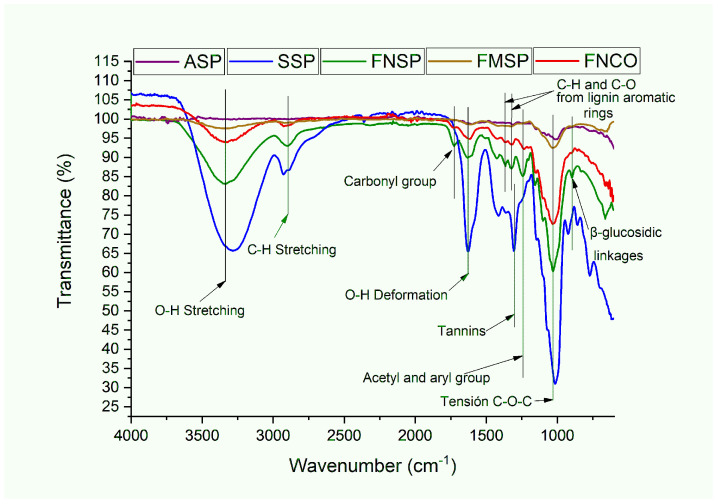
Spectrograms of materials from plantain pseudostems.

The materials from the pseudostem that we evaluated were starch, sap, native lignocellulosic fibers from the core, and native and modified fibers from the sheaths (see Figure 2). Typical signals (O-H bond stress vibrations, C-H strain, O-H strain, and C-O-C strain) with different transmittance intensities were evident in each sample. In the tension signal corresponding to O-H bonds located between 3600 and 3000 cm^−1^ [9], higher intensity was identified in the sap, followed by the native fiber coming from the sheaths, relating its higher capacity for water absorption due to the presence of tannins, phenolic compounds, flavonoids, aromatic amino acids, reducing sugars (glucose, fructose, xylose, arabinose, galactose), and sucrose [1,15,16,51,52,53,54]. Similarly, Basak et al., 2015 [55], reported the presence of inorganic salts from the peak formation between 800 and 1300 cm^−1^, finding phosphate groups between 1100 and 1000 cm^−1^, magnesium chloride and potassium chloride salts between 1176 and 873 cm^−1^, and sodium phosphate at 1000 cm^−1^. However, Gonultas et al., 2012 [56], mentioned that the band between 1500 and 950 cm^−1^ is related to the presence of tannins. Balakrishnan et al., 2021 [9], mentioned that apart from relating the water absorption capacity, C=C bonds belonging to aromatic groups were identified in the signal located between 1600 and 1450 cm^−1^, where the plantain sap possibly presents the highest concentration of components with antioxidant characteristics.

Similarly, the sap presented the highest intensity in the tension signal in the C-H bonds at 2886 cm^−1^, which was possibly related to the higher concentration of aldehydes (H-C=O) present in its structure [9]. The intensity of the previous signal (C-H) identified in the sheath fibers relates to the presence of hemicellulose [57,58], identifying a reduction in its magnitude in the steam-exploded fibers. Regarding other structural changes between native and steam-exploded fibers, differences are evident in the following set of signals identifying the presence of lignin and hemicellulose: between 1740 and 1730 cm^−1^, a characteristic peak of carbonyl groups identifying hemicellulose and pectin was evident [5,9,23,34], the C-H and C-O groups belonging to the aromatic rings of hemicellulose and lignin being represented in peaks between 1360 and 1320 cm^−1^ [57], the presence of acetyl and aryl groups (C=O and C-O) belonging to the structures of hemicellulose and lignin at 1242 cm^−1^ [23,34], and β-glucosidic bonds between cellulose and hemicellulose at values below 1000 cm^−1^ [5]. Evidence of these was mainly seen in the native lignocellulosic fibers coming from the sheaths and core, keeping the highest intensity in the corresponding ones from the sheaths due to a possible higher concentration of hemicellulose and lignin. According to Figure 2, the action of the steam explosion generated a reduction in the intensity of the peaks, altering the chemical composition of the fibers [23]. It is possible that the physical surface modification contributed to the removal of some portions of lignin and hemicellulose This behavior is similar to that reported with rice straw subjected to steam explosion [58,59], despite employing higher pressure (1.7 MPa) and temperature (205 °C). On the other hand, Pereira-Marques et al., 2021 [60], reported the reduction in the signal magnitude at 1740 cm^−1^, associating it with a reduction in hemicellulose presence in sugarcane bagasse fibers when employing a temperature of 168 °C. Zhao et al., 2023 [61], when processing bamboo fibers at 2 MPa for 4 min, did not evidence the formation of new functional groups, although a reduction in the intensity of the signals present was generated.

Regarding the band located at values close to 1600 cm^−1^ that identifies the O-H strain vibrations, the degree of hygroscopicity of the material is related [22] and is dependent on the greater water absorption capacity in the plantain sap among the group of materials found in the pseudostem. On the other hand, the native lignocellulosic fibers from the sheaths have a higher degree of hygroscopicity compared to those found in the core and the fibers modified on their surface by steam explosion. In contrast, the starch from the core produced the lowest value in this capacity compared to the group. Periyasamy et al., 2022 [24], reported that steam explosion generates the hydrolysis of hemicellulose, contributing to the increase in crystallinity in cellulose from the transformation of the amorphous phase, while Vishnu Vardhini et al., 2018 [49], described the surface modification of fibers with changes in their diameters, structure, chemical composition, surface morphology, crystallinity, and their water absorption capacity due to the removal of hemicellulose in their structure. In this case, considering the results found in the chemical composition, FT-IR identified the detachment of some portions of hemicellulose, causing a lower concentration in the steam-exploded fibers. Another possible explanation for the lower hygroscopicity of the modified sheath fibers is based on the fact that, initially, the sap and the fibers present a direct contact in the natural structure of the pseudostem sheaths, with some hydrophilic components of the sap (phosphates, minerals, and sugars) remaining adhered to the surface of the native fibers, which are subsequently extracted in high proportion when the fibers are subjected to the steam explosion process.

### 4.4. Scanning Electron Microscopy (SEM)

**Figure 3:**
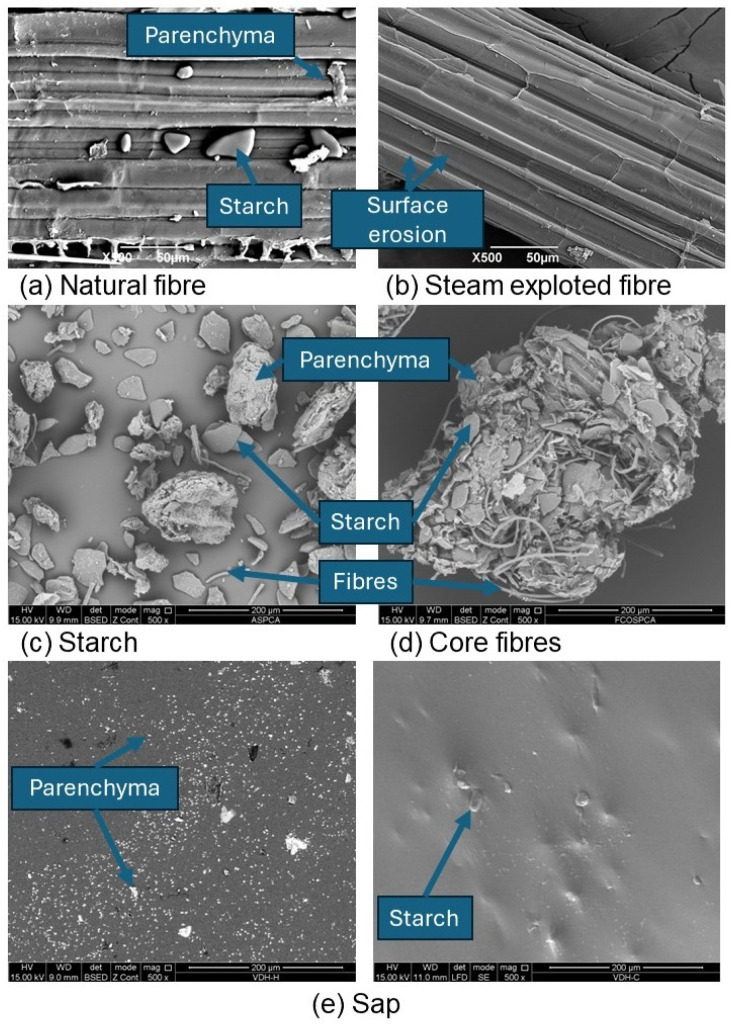
Micrographs of native and modified sheath fibers.

The native fibers from the sheaths presented a surface with a higher degree of impurities compared to the fibers subjected to steam explosion (see Figure 3a,b), finding starch granules and fragments of parenchyma adhered to the fiber surface. The presence of the non-lignocellulosic plant material can affect the use of lignocellulosic fibers when used in different applications, such as papermaking and composites [12], as it reduces the interfacial adhesion with a polymeric matrix. The action of high-temperature water vapor contributed to removing the particles, and a cleaner surface was evidenced (see Figure 3b). Pang et al., 2024 [58], identified another behavior related to the formation of pores in the lignocellulosic fibers of wheat bran after being subjected to steam explosion with a pressure of 0.5 MPa, which was a more aggressive condition compared to the exposure of the sheath fibers from the plantain pseudostem, without evidence of very pronounced porosities in its structure. However, it was possible to identify some reduced cavities and microfibrils of greater relief on the surface due to erosion generated by the action of water vapor. Chen et al., 2011 [59], and Zhao et al., 2023 [61], reported that surface erosion caused by steam explosion is related to the removal of some parenchyma cells adhered to the surface, hemicellulose, and lignin. Figure 3c,d shows two types of by-products from the core, starch, and native fibers, identifying their shapes, dimensions, and degree of purity at a qualitative level. The shape of the starch granules from the core is elongated and irregular, presenting an average diameter of 39.55 + 8.69 µm with the existence of parenchyma particles and fibers, differing from the starches coming from the pulp and peel, by maintaining elongated shapes with oval ends of greater definition, which is a concept also identified by Mohd Ali et al., 2021 [14], when evaluating the morphology of starch granules belonging to the banana pseudostem. Shantha and Siddappa, 1970 [39], found that granules originating from the pseudostem have a diameter greater than that found in pulp starches, with values between 23.91 and 38.97 µm [62,63]. The presence of parenchyma particles and fibers in the core starch contributes to its low degree of purity and relates to the low hygroscopicity of FT-IR. In the case of fibers, the procedure used for their isolation contributed to the formation of agglomerates formed by fibrils, with an average diameter of 3.69 + 0.65 µm, entangled with starch granules and parenchyma particles.

Meanwhile, the plantain sap (see Figure 3e) presented a smooth and soft surface with impurities that are possibly related to the residual parenchyma and starch granules. In the case of the parenchyma, two types of particles were evidenced, the first being of smaller size (2.85 + 0.69 µm) with cylindrical and oval geometries, while the larger ones (18.00 + 7.48 µm) presented an asymmetrical shape.

### 4.5. Diameter and Linear Density of Plantain Fibers and Yarns

The native lignocellulosic fibers extracted from the plantain pseudostem had an average diameter of 293.20 µm, which is higher than that reported by Balakrishnan et al., 2021 [9]. When characterizing fibers from two varieties of banana (puwalu and ambun), they obtained diameters between 80.04 and 123 µm. Jayaprabha et al., 2011 [12], identified a variation in the diameter of the native fibers of the Nendran variety of banana, finding values between 50 and 250 µm, highlighting that the fibers with the largest diameter are found in the sheaths located on the external part of the plantain pseudostem structure. In the case of fibers from plantain, diameter values of up to 650 µm can be obtained [10]. Accordingly, the properties of lignocellulosic fibers have shown differences in their magnitudes when considering the botanical source, variety, soil quality, and agroclimatic conditions [12]. By subjecting these fibers to surface modification using steam explosion, a reduction in diameter was achieved (see Table 3), a physical behavior identical to that reported by Deepa et al., 2011 [64], and Abraham et al., 2011 [65], when subjecting fibers in such surface modification. Danso, 2021 [10], highlights that reducing the diameter can increase the fiber’s aspect ratio (L/D), enhancing the mechanical properties when these fibers are mixed with a polymeric matrix to form a composite material. Jayaprabha et al., 2011 [12], mentioned that reducing fiber diameter reduces structural defects, providing a greater macromolecular arrangement in the fibers due to the release of hemicellulose and lignin particles in the fibers.

When the two types of lignocellulosic fibers of the sheath (native and steam-exploded) were available, their respective yarns were elaborated, identifying that the conformation of the native yarns could be made from the shorter length of the ends of the fibers for their entanglement with new fibers, allowing the prolongation of the length of the yarn and avoiding its breakage as it is wound on the bobbin of the spinning machine.. When using steam-exploded fibers, an opposite behavior was identified to produce the yarn since a greater length of the fibers’ ends was required for such mechanical action. As a result, the native yarns achieved lower values in diameter and linear density (see Table 3). Possibly, the removal of residual parenchyma and starch, including the detachment of low portions of lignin and hemicellulose on the surface of the fibers from the pseudostem, generated a decrease in surface roughness, requiring the fibers to be longer in order to achieve entanglement and yarn formation.

### 4.6. Tensile Test

Native lignocellulosic fibers extracted from plantain pseudostems presented a σ_max_ of 232.40 MPa, an E of 9.35 GPa, and an ε of 3.99%. When compared with native fibers from Nendran banana pseudostems, Jayaprabha et al., 2011 [12], reported a σ_max_ of 244.03 MPa with diameters close to 200 µm, while the same type of fibers with diameters greater than 250 µm generated values of 182.33 MPa. As for ε, independent of fiber diameter, its values ranged from 2.04 to 2.28%. Kenned et al., 2020 [21], reported a σ_max_ of 412.5 + 46.7 MPa in Nendran variety banana fibers, including a 138.0 + 0.07 µm diameter and an ε of 27.89%, while Danso, 2021 [10], reported a diameter of 650 µm and a σ_max_ of 59.9 MPa in plantain fibers. According to the above, some σ_max_ values are obtained, like those in banana and plantain. In musaceae, a reduction in said property to tension can be obtained as the fiber diameter increases. However, there is also a second parameter that defines the tensile properties in lignocellulosic fibers, which is the cellulose content, where the high σ_max_ in tension reported by Kenned et al., 2020 [21], is related to the high cellulose content (71.08%), low lignin content (7.67%), and a low microfibrillar angle (11°), whereas the contents of the two macromolecules mentioned above were opposite to those obtained in the native plantain fibers under study. A similar behavior was evidenced in what was reported by Venegas et al., 2022 [47], when using fibers from the pseudostem of the plantain variety Hartón, reporting a σ_max_ at a tension of 440.30 MPa and an E of 15.67 GPa, since in their chemical composition, they contained 69.49% cellulose and 13.17% lignin. The effect of steam explosion on the fibers from the pseudostem sheaths generated an increase in the σ_max_ and ε while reducing the E. This was possibly due to removing portions of hemicellulose and lignin from the fibers, which increased cellulose content, supporting greater applied stress. On the other hand, when these macromolecules were removed from the fibers, spacing between the cellulose, lignin, and hemicellulose chains was generated, which increased the strain capacity and intermolecular movements. At the same time, the reduction in E relates to the decrease in bonds formed in the fiber by removing the components above from its structure since, through FT-IR, the absence of β-glucosidic bonds was identified in the steam-exploded fibers, which were those responsible for forming the bond between cellulose and hemicellulose in their native states. At the time of obtaining the respective yarns, considering the differences in their diameters and linear densities, a higher tenacity was evidenced in the native yarns (see Table 3) due to the possible generation of shear stresses of greater magnitude between the surfaces of the fibers that make it up. Gañán et al., 2008 [5], reported the tenacity and ε of the native fibers coming from the sheaths of the plantain Dominico Hartón with values of 0.47 + 0.12 N/Tex and 1.9 + 0.8%, respectively, which are lower than the ones we obtained.

### 4.7. Thermal Properties of Fibers, Starch, and Sap from Plantain Pseudostems

#### 4.7.1. Thermogravimetric Analysis (TGA)

**Figure 4:**
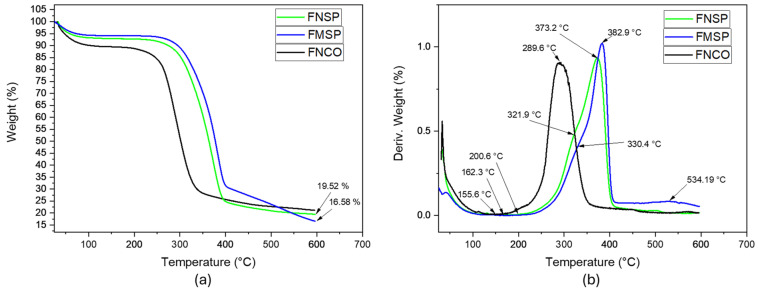
TGA (**a**) and DTGA (**b**) thermograms of lignocellulosic fibers from plantain pseudostems.

In the present thermal analysis, the signals or peaks generated by DTGA should be considered, identifying a sequence in thermal degradation, starting with hemicellulose between 178 and 300 °C, followed by cellulose between 200 and 400 °C and ending with lignin between 420 and 700 °C [22,28]. In the case of the evaluated fibers, it was determined that those in the native state coming from the core presented an onset of thermal degradation at 155 °C, which was lower than that generated in the native fibers of the sheaths, with a value of 162.3 °C (see Figure 4). Even though the fibers coming from the core presented lower lignin content and such pseudostem structure may contain the highest proportion of pectin and starch compared with the pseudostem sheaths [14,15], it is possible that such lignocellulosic fibers are mixed with the components above that confer lower thermal stability. After subjecting the native fibers from the sheaths to steam explosion, an increase in the onset of thermal degradation was generated, passing to 200.6 °C. This behavior is similar to that evidenced by Chen et al., 2011 [59], who used rice bagasse fibers in a steam explosion and increased the thermal degradation temperature from 226.9 °C in its native state to 257.7 °C after modification. Another thermal parameter that can be used is the peaks, or shoulders, generated in the DTGA, identifying a signal between 309 and 350 °C that possibly relates the amount of hemicellulose present in the lignocellulosic fibers coming from the sheaths, evidencing lower magnitude in the steam-exploded fibers with a value of 330.4 °C, while in the native fibers, its value was 321.9 °C (see Figure 4b). The second signal identified was related to the T_dpeak_ of 50% of the cellulose, reporting a value of 289.6 °C in the core fiber, 373.2 °C in the native fiber from the Selleck, and 382.9 °C in the steam-exploded fibers from the Selleck. In the third characteristic signal of lignin identified above 450 °C, less thermal stability of this macromolecule was evidenced in the steam-exploded fibers of the sourdough when compared to the respective native fiber since they maintained a mass of 16.58 and 19.52% at 600 °C, respectively (see Figure 4a).

**Figure 5:**
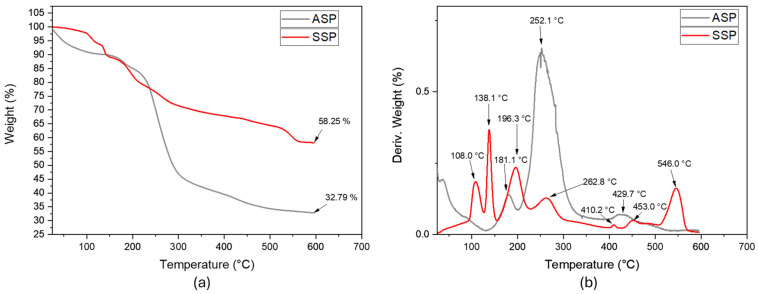
(**a**) TGA and (**b**) DTGA thermograms of plantain starch and sap.

In the case of core starch, a T_dpeak_ of 252.1 °C was identified, which was lower than that presented by starches coming from other plantain by-products (see Figure 5), since starches belonging to plantain pulp and peel presented values between 280 and 330 °C [66,67]. Another signal was identified at 429.7 °C, possibly relating to lignin coming from the parenchyma and fibers identified in the SEM. While the sap recorded seven signals at 108.0, 138.1, 196.3, 262.8, 410.2, 429.7, and 546 °C, which are associated with the presence of different types of molecules relating to the presence of moisture (108 °C), volatile components (138.1 °C), terpenes (196.3 °C) and phenolic compounds (between 262.8 and 429.7 °C) [68,69,70,71]. This by-product has the highest degree of thermal stability until reaching 600 °C, as it retains 58.25% of its mass as ash, which might be due to its high mineral content. Basak et al., 2015 [72], Basak et al., 2015 [73], Basak et al., 2016 [18], Basak et al., 2015 [55], and Gupta et al., 2019 [15], reported the preservation of 40% of the mass of banana sap when exposed to 500 °C under inert conditions (presence of nitrogen) due to the presence of some minerals, such as magnesium, phosphorus, potassium, sodium, silicon, chlorine, calcium, and aluminum. This was examined through the use of an energy dispersive X-ray (SEM-EDX) and the results recommended the use of the sap as a flame retardant, using it as a coating on fabrics, paper, and paperboard.

#### 4.7.2. Differential Scanning Calorimetry (DSC)

**Figure 6:**
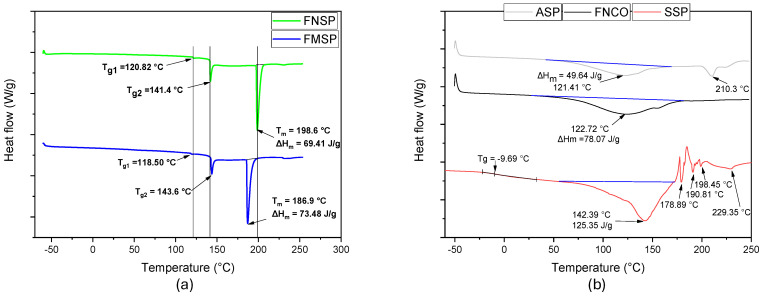
Thermograms DSC of (**a**) native and modified sheaths fibers; (**b**) core fibers, core starch, and core sap.

Considering that the steam explosion operation was performed at 120 °C, and that the onset of thermal degradation of the native and modified fibers was evidenced at 162.3 and 200.6 °C, respectively, as reported by the TGA, the signals evidenced in the DSC thermograms (see Figure 6) are possibly related to the Tg1 of hemicellulose, the Tg2 of lignin, and the Tm of lignin, hemicellulose, and amorphous cellulose. Steam explosion applied to lignocellulosic fiber from pseudostem sheaths showed a reduction in Tg1 corresponding to hemicellulose from 120.82 to 118.50 °C, relating this behavior to the possible reduction in hemicellulose content on the surface of lignocellulosic fibers [74]. Hemicellulose hydrolyzes and solubilizes in water after high pressure and temperature exposure, forming lower molecular weight molecules, such as glucose and xylose [29]. In the second Tg (Tg2) located at 141.4 °C, the presence of lignin is related, since Deepa et al., 2011 [64], and Ibrahim et al., 2010 [75], reported the Tg of lignin at 142 °C, while Müssig, 2010 [27], and Poletto, 2017 [76], mentioned that the Tg of lignin can be found between 90 and 180 °C, depending on the plant species providing such fibers. Upon undergoing physical modification, an increase in the second order transition of the fibers was generated, going from 141.4 to 143.6 °C, due to the removal of a reduced portion of lignin in the fibers, as indicated by the Klason test and research conducted by Agbor et al., 2011 [29], and Mosier et al., 2005 [37]. Kong et al., 2017 [77], mentioned that the increase in Tg relates to an increase in interactions between cellulose macromolecular chains and the formation of hydrogen bridges, leading to increased molecular restraint in the fibers. The above behavior can be corroborated by the higher σ_max_ in the steam-exploded fibers when subjected to the tensile test. In the third transition identified in the native fiber from the pseudostem stock, the Tm identified at 198.6 °C possibly relates to the presence of hemicellulose and lignin. This is because Kabir et al., 2012 [78], reported the presence of hemicellulose Tm between 10 and 200 °C, while Müssig, 2010 [27], reported the Tm of lignin at 170 °C. The effect generated by the steam explosion separated limited portions of lignin and hemicellulose from the lignocellulosic fibers, contributing to a reduction in the Tm value at 186.9 °C; however, when comparing the ΔHm of the exploded fiber with the native fiber, a higher value was obtained, which was due to the possible generation of amorphous cellulose [79], finding values of the latter macromolecule at values close to 190 °C after subjecting the fibers to physical and/or chemical modification. In the case of core starch, a Tm of 121.41 °C and a ΔHm of 49.64 J/g were identified, which were lower values compared to those reported for pulp and peel starches from plantain. In the core fibers, a Tm at 122.72 °C is identified, relating to the presence of remaining starch, but with a higher ΔHm (see Figure 6), requiring higher thermal energy to melt the starch granules found within the agglomerations identified through SEM. Finally, the sap presented a Tg below 0 °C, relating to a structure with a flexible behavior when employed in applications above −9.69 °C, without considering its high hygroscopicity, which may confer adhesiveness. However, below the temperature above, its structure has a rigid behavior. With increasing temperature in the sap, four endothermic peaks were identified, relating to the possible presence of a crystalline phase (larger peak) that melts at 142.39 °C with a ΔHm of 125.35 J/g. The other peaks probably correspond to other components present in the sap.

## 5. Conclusions

The processing of pseudostems has a high potential for producing biobased materials due to the identification of two types of lignocellulosic fibers, starch, and sap, achieving the utilization of approximately 4% of its biomass using a mechanical paddle shredder, drying, milling, and screening.

The steam explosion slightly increased cellulose and reduced lignin content, according to chemical composition (Tappi T-203), DSC, and TGA. In the case of hemicellulose, according to the chemical composition, no quantitative differentiation was achieved (Tappi T-203). However, FT-IR spectroscopy, TGA, and DSC identified the reduction in its content in the steam-exploded fibers at a qualitative level. Removing hemicellulose and lignin from the fibers contributed to an increase in σ_max_ and ε; however, it reduced E.

It was possible to produce yarn through native and steam-exploded lignocellulosic fibers, differentiating those of native state in the processing and their mechanical properties since greater adherence was identified between the long fibers that comprised it, granting lower linear density and greater tenacity. Sap presents a wide diversity of components, such as tannins, fibers, and minerals, as identified in the TGA and DSC. The high mineral content allows it to perform in applications requiring high thermal stability.

Regarding its physicochemical and thermal properties, according to FT-IR and DSC, it presented the highest degree of hygroscopicity, high adhesiveness, and flexibility at temperatures above 0 °C. In the case of the fibers belonging to the core, the lowest thermal stability was observed. In contrast, the steam-exploded fibers of the sheaths showed the highest thermal stability, the latter being recommended for high-temperature applications up to 200.6 °C.

## Figures and Tables

**Table 1:** Composition of sheaths and core in pseudostems.

Pseudostem By-Products (%)			
Stocking		Core	
80.85 ± 0.71		19.14 ± 0.70	
Lignocellulosic fibers	Sap	Lignocellulosic fibers	Starch
2.20 ± 0.16 ^1^	2.12 ± 0.46 ^1^	6.39 ± 0.53 ^2^	12.81 ± 0.47 ^2^

Yield on a wet basis. Yield concerning fresh sheaths ^1^. Yield concerning fresh core ^2^.

**Table 2:** Lignin content in native and modified plantain fibers.

By-Product	Component	Holocellulose (%)	Cellulose (%)	Hemicellulose (%)	Lignin (%)	Ashes (%)
sheaths	Native fiber	68.64	54.72 ± 2.60	13.92 ± 2.50	27.68 ± 5.1	1.84 ± 0.01
	Steam-exploded fiber	69.30	54.66 ± 0.48	14.64 ± 5.90	26.78 ± 3.9	1.96 ± 0.03
	Alkalized fiber	-	-	-	16.76 ± 3.5	-
Core	Native fiber	77.36	-	-	9.02 ± 2.5	17.62 ± 0.14

**Table 3:** Physical properties, stress, and tenacity in plantain-based fibers and yarns.

Propiedades Físicas y Mecánicas	Native Fiber	Steam-Exploded Fiber	Native Yarn	Steam-Exploded Yarn
Diameter (µm)	293.20 ± 47.57	168.58 ± 36.13	1465.36 ± 195.40	1840.51 ± 239.92
σ (MPa)	232.40 ± 88.86	246.00 ± 66.93	-	-
E (GPa)	9.35 ± 3.50	6.47 ± 2.41	-	-
ε (%)	3.99 ± 3.54	11.70 ± 7.30	-	-
Linear density (Tex)	1.73 ± 0.58	1.27 ± 0.41	4.18 ± 1.96	4.25 ± 1.12
Toughness (N/Tex)	8.42 ± 2.33	4.25 ± 1.54	11.82 ± 3.51	9.56 ± 2.18

## Data Availability

Data are contained within the article.
